# Transactions

**Published:** 1878-09

**Authors:** 


### TRANSACTIONS.

The proceedings of the joint meeting of the American Dental Convention, the Southern Dental Association and the Dental Association of the State of Maryland and District of Columbia, held at Oakland, Maryland, in August, 1877, is at hand. It contains much valuable matter that every member of the dental profession would, or ought to, be interesting in reading.

There is always much of interest and value in the proceedings of all of our societies. The thoughts and ideas of our advanced thinkers there crop out, and are often presented in sharper outline than elsewhere.

Every dentist who desires to keep up with the times, should procure copies of the transactions of all our societies, so far as they may be published.

